# How risk communication affects public trust in government: the moderating role of policy expectations

**DOI:** 10.3389/fpubh.2025.1557786

**Published:** 2025-05-14

**Authors:** Nuoxue Li, Weixi Zeng, Susu Yin, Lixia Zhao

**Affiliations:** University of Electronic Science and Technology, Chengdu City, Sichuan Province, China

**Keywords:** conspiracy narratives, risk perception, risk communication, policy expectations, government trust

## Abstract

**Introduction:**

Conspiracy narratives are a prevalent narrative framework in risk communication, often provoking public fear and defensive reactions, challenging the healthy interaction between governments and the public in social governance.

**Method:**

Through two survey experiments, this study explores the effects of conspiracy narratives on public trust in government and the moderating role of policy expectations.

**Results:**

In Study 1 (*N* = 119), conspiracy narratives increased public perceptions of and concerns about the risks of genetically modified (GM) foods. As a result, the public was more likely to expect the government to adopt a strictly restrictive control policy on GM foods. Study 2 (*N* = 119) further reveals that public trust in the government increases when the public perceives the government as implementing a restrictive policy on GM foods. Conversely, public trust declines if the government is perceived to promote GM foods actively. Policy satisfaction plays a fully mediating role in this process.

**Discussion:**

The study reveals the influence mechanism of conspiracy narratives on government trust, offering both a theoretical basis and practical recommendations for effective government communication and the development of harmonious government-public relations.

## 1 Introduction

The theory of risk society reveals a fundamental paradox: while creating modern civilization, technological development systematically produces new types of risks beyond traditional perceptions (1–3). This mechanism of risk production presents two distinctive features in the context of the new industrial revolution: first, technological risks such as artificial intelligence and gene editing are irreversible and globally diffuse (4–6); and second, the mechanism of risk perception formation is increasingly subject to structural distortions in risk information dissemination and communication (7, 8). Numerous empirical studies have shown that the public’s subjective assessment of technology risk generally deviates from objective data (8, 9), resulting in significant cognitive disembedding. This disembedding not only stems from the cognitive limitations of information receivers (10) but also from the framing effect of risk narratives: when technological risks are reconstructed by specific narrative strategies (e.g., conspiracy theories, technological determinism), the public’s risk judgments will be systematically skewed (11–13). The accumulation of such cognitive biases ultimately leads to risk communication falling into the trap of “narrative-dominated rationality” (14).

The effectiveness of risk communication in shaping public risk perceptions has intensified with the development of digital media. Through algorithmic recommendations and social communication, new media platforms make conspiracy narratives more accessible to groups that lack critical judgment. These narratives often capitalize on cognitive biases and lack of evidence to reduce complex social events to a conspiracy framework that is “manipulated by a hidden group” (7, 15), with typical examples including NASA’s moon landing fakery (16), and Princess Diana’s assassination conspiracy theories (17), which significantly amplify the public’s perception of technological risk through the construction of false causal relationships (18, 19). Notably, this narrative frame not only distorts individual perceptions but also creates collective memory contamination through social networks. When conspiracy narratives gain group acceptance, their erosion of the social trust system has a multiplier effect (15, 20).

When the public develops risk perceptions, it tends to develop fear and uncertainty (21), and in turn expects the government to take risk management measures (22, 23). It is worth noting that risk governance models differ significantly across countries and regions: North American countries, represented by the United States and Canada, tend to adopt opportunistic risk management strategies (22), while the European Union (EU) generally implements more prudent regulatory policies (24, 25). This is particularly manifest in the Chinese context, where public expectations of government risk management capabilities are significantly higher than in Western countries (26), influenced by the cultural trait of high power distance (27). This phenomenon leads to two key research questions: first, how would conspiracy narratives shape public policy expectations by influencing risk perceptions? Second, when the government’s actual policy preferences deviate from public expectations, how does this affect public trust in the government?

Important gaps remain in the existing literature regarding risk perception research. While studies have explored the impact of conspiracy theories on public health and environmental topics (28, 29), there is still a lack of in-depth exploration of the mechanisms of risk narratives related to the emerging field of biotechnology, especially genetic engineering. More importantly, although scholars have recognized the critical role of policy expectations in connecting risk perception and public trust in the government (26), existing research has yet to systematically explain how risk narratives ultimately affect the dynamic process of political trust through policy expectations.

Based on the above research gaps, the selection of genetically modified (GM) technology as a research object in this study has special theoretical value. GM food, which is directly related to daily dietary safety, is very likely to trigger sensitive reactions from the public (30, 31). This sensitivity stems from a fundamental contradiction: on the one hand, the enormous industrial potential offered by biotechnological innovations, and on the other hand, the public’s deep-seated concern about “genetic modification” (32–35). It is this ambivalence that makes GM technology an ideal case for examining the relationship between risk perception and public trust in the government.

This study designed two progressive experimental studies to explore this issue in depth. Experiment 1 breaks through the limitations of traditional studies that focus on technical rationality narratives (9, 10), and focuses on the reinforcing effect of conspiracy narratives on GM risk perceptions, and how this effect is transmitted through policy expectations. On this basis, Experiment 2 further constructs a complete chain model of “narrative-perception-expectation-trust.” And empirically examines the dynamics of policy satisfaction and institutional trust when the actual government policy deviates from the public’s expectation by modeling the Chinese government’s unique prudent policy stance (36).

This research design has important innovation value. From the theoretical research aspect, firstly, it expands the boundaries of applying risk society theory in authoritarian governance; secondly, it provides new empirical evidence for understanding the mechanism of political trust in technological controversies through cross-cultural comparison. On the practical level, the findings will provide an important theoretical basis for government departments to construct a more effective risk governance paradigm in the new media era, especially when dealing with highly controversial technological topics such as genetic modification, which can help policymakers better balance the relationship between technological innovation and public trust.

## 2 Literature review and research hypotheses

### 2.1 The role of conspiracy narratives in shaping risk perception

A narrative is a structured expression integrating events and characters to convey a specific topic (36). Narratives play a critical role in shaping audiences’ cognitive and affective responses (37). Risk perception, defined as an individual or group’s subjective judgment about the likelihood and potential harm of a risk source, is a key concept in understanding public responses to emerging threats (38). Prior research demonstrates that narrative framing significantly influences public risk perceptions and emotional responses in domains such as health and environmental risks (18, 30).

With the rapid advancement of technologies like genetically modified bioengineering, nanomaterials, and autonomous driving, technological risk has emerged as a prominent focus of risk research (4–6). For instance, risks such as privacy breaches, ethical dilemmas, and value conflicts are often associated with discussions of generative AI technologies among college students (39). While technological risks typically emerge in the present, their impacts often unfold in the future (57), making them a central feature of the “risk society” and intensifying public concerns about such risks (3).

Against the backdrop of international tensions and rapid technological iteration, conspiracy theories have proliferated as a pervasive narrative framework (7). Conspiracy narratives attribute significant political and social events to covert schemes orchestrated by influential entities (15, 40). The widespread adoption of new media platforms has made these narratives easily accessible to a broader audience, particularly to individuals with limited critical judgment (15). For example, during the COVID-19 pandemic, conspiracy narratives portraying vaccinators as victims fueled public paranoia, increased vaccine hesitancy, and amplified risk perception (19, 20).When confronting products involving novel technologies, such as genetically modified (GM) foods, the general public often lacks the specialized knowledge needed to assess risks. This knowledge gap is rarely bridged through formal education (6, 41).

Thus, narrative framing has become an influential informal mechanism for shaping public perceptions of risk (18). To examine this phenomenon, this study contrasts technological risk narratives, conspiracy narratives, and neutral materials lacking narrative orientation. Based on this, we hypothesize:

*H1*: Different narrative frames exert distinct effects on risk perception during risk communication.

Conspiracy narratives, in particular, elicit unknown and uncontrollable emotional reactions compared to technological risk narratives (15). They tend to incorporate strong emotional appeals and attributional inferences, provoking public anxiety and unease (40, 42). Such narratives often extend beyond technological risks, linking them to external threats posed by specific nations or groups, thereby amplifying fears about exploitation and harm to national interests (43, 44). Consequently, conspiracy narratives are expected to heighten public risk perception more effectively than technological risk narratives. Thus, we hypothesize:

*H2*: Conspiracy narratives in risk communication lead to stronger public perceptions of social risk compared to technological risk narratives.

### 2.2 Risk perception and public policy expectations

Public expectations regarding government actions and policies are a crucial component of behavioral public management (27, 45). Policy expectations represent the public’s beliefs about the government’s ability to address specific risks through policy formulation and implementation. Rooted in principal-agent theory, individuals, as principals, form expectations about government actions and hope for alignment with their preferences. These expectations can range from rational to irrational and optimistic to pessimistic (46).

Risk perception—the assessment of the likelihood and potential consequences of a hazard—plays a key role in shaping policy expectations (21). During periods of uncertainty, individuals often rely on political systems and institutions to manage their fears and threats (47). However, the extent of this reliance varies across political and cultural contexts. For instance, Hofstede highlights the collectivist tendencies and uncertainty avoidance in Chinese culture, contrasting with the individualistic, libertarian ideals of the United States (48). This cultural divergence was evident during the COVID-19 pandemic: Chinese microbloggers predominantly exhibited trust in authorities and positive attitudes toward vaccination, while American Twitter users often expressed personal experiences and vaccine skepticism (49).

Under the influence of Chinese Confucianism and Legalism, the government’s management model combines authority with benevolence, fostering what some scholars describe as a “mission-responsibility” relationship between the government and its citizens (58). The collectivist and high power-distance tendencies of Chinese culture contribute to greater public reliance on the government for risk management (26). Accordingly, we hypothesize:

*H3*: Conspiracy narratives increase public perceptions of social risk, heightening public expectations for stricter government control policies.

### 2.3 Policy satisfaction and trust in government

Trust in government is a fundamental topic in political science, reflecting the public’s evaluations and emotional orientations toward the political system, institutions, and processes (26). Various factors influence trust in government, including socioeconomic conditions, perceptions of fairness, policy satisfaction, and policy expectations (50, 51, 59). Trust in government often hinges on whether the public perceives that government actions align with their expectations (52, 53).

Research suggests that when public policy outcomes fail to meet expectations, dissatisfaction can lead to political cynicism and erode trust in government (54). Conspiracy narratives, by associating risks with specific groups or nations, may further intensify public scrutiny of policy alignment (55). When government policies deviate from these expectations, policy dissatisfaction may reduce public trust. To examine this, Experiment 2 considers two policy orientations (“strict restriction” vs. “active promotion”) and assesses the influence of conspiracy narratives. We hypothesize:

*H4*: Conspiracy narratives in which government policies to restrict GM foods trigger higher government trust than policies to promote them.

*H5*: Conspiracy narratives in which government policies of restriction on enemy GM technology significantly increase public trust over cooperative policies.

*H6*: Public policy satisfaction fully mediates policy orientation and government trust.

*H7*: Public policy expectations mediate the relationship between government policy orientation and public policy satisfaction.

**Figure 1:**
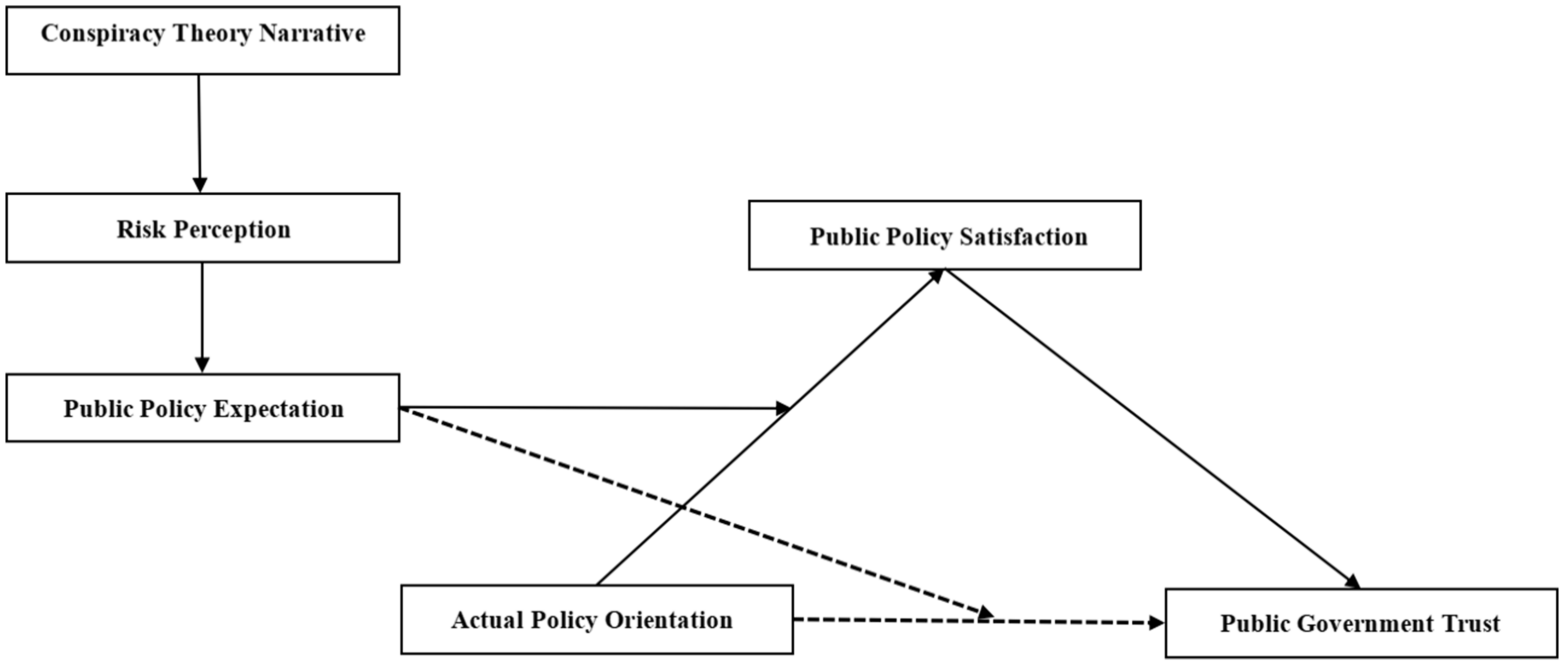
The model diagram illustrates the relationships between variables.

This study investigates three primary variables within the proposed framework (Figure 1): (1) the impact of narrative framing on public risk perception. (2) The relationship between risk perception and public policy expectations. (3) The moderating role of public policy expectations in the relationship between policy orientation, policy satisfaction, and trust in government. (4) The mediating role of policy satisfaction in linking policy orientation to public trust in government.

## 3 Experiment 1: the effect of narrative frame and risk perception on policy expectations

The purpose of Study 1 is to initially investigate the effects of different risk narrative frames on public risk perceptions and policy expectations, especially whether conspiracy narratives significantly enhance public risk perceptions and policy expectations. In this experiment, a situational approach was adopted, in which subjects were asked to read scenarios about GM food (technology) under different risk narrative frameworks and report their risk perceptions and policy expectations in order to examine the differences in risk perceptions and policy expectations among the public in different risk narrative frameworks.

### 3.1 Subjects

The sample size required for the experiment was estimated according to G*Power 3.1. For the test used in Experiment 1, the required sample size should be no less than 159 for setting the significance level (*α* = 0.05), the desired efficacy value (1 − *β* = 0.80), and guaranteeing a medium effect size (*f* = 0.25). Four hundred twenty subjects were recruited offline for this study, and data with incomplete information or extreme attitudes [(e.g., selecting “1” for all items) were excluded.], and finally obtained 369 valid data, with a recall rate of 87.85%. Among them are the “conspiracy narrative” group (*n* = 119), the “technological risk narrative” group (*n* = 120), and the “neutral narrative” group (*n* = 130). Regarding the selection of subjects, the college student group, Internet Aborigines, has an average daily online time of more than 8 h and belongs to the group with the highest Internet activity. In addition, as a group with high-end education, their rational, analytical thinking, and cognitive reflection abilities are significantly higher than those of the general group. This cognitive characteristic makes them more immune to information bias. Suppose significant cognitive bias can still be observed in the experiment, according to the principle of ecological validity extrapolation. In that case, its effect strength may produce a “floor effect” in the general Internet users group, i.e., showing more significant bias characteristics. The present study was conducted with college students through an offline questionnaire. Two hundred and forty of the 369 participants were male, accounting for 65% of the sample. 68.3% of the participants were male and 68.3% were male. In addition, 68.3% of the participants were majoring in science and technology. Table 1 shows the detailed demographic information of the participants, and all the information was paid accordingly after the completion of the study.

**Table 1:** Demographic information of subjects (study 1).

(*N* = 369)	*N*	%
Gender		
Male	240	65.0%
Female	129	35.0%
Household registration		
Countryside	116	31.4%
Cities and towns	116	31.4%
Small or medium size city	85	23.0%
Large cities	52	14.1%
Major		
Science and Engineering	252	68.3%
Social Sciences (Economics, Law and Education)	81	22.0%
Humanities (Literature, History and Philosophy)	29	7.9%
Art and Physical education	7	1.9%

### 3.2 Experimental materials

In order to ensure the authenticity and readability of the material, this study combines opinions against GM food (technology) through online and in-depth personal interviews to categorize the reasons for opposing GM as follows: (1) Potential threats of GM technology: ecological and environmental threats, threats to full human health, and other hidden dangers. (2) GM is a weapon of the West to persecute the Chinese people: the core technology is in the hands of the West, and it is a new type of biochemical weapon that will make China die. The two main categories of reasons for the appeal were analyzed. The rumor part was eliminated to extract two important reasons that may affect the people’s trust in the government, i.e., the existence of the threat from the technological risk of GM food (technology) and the existence of the threat of artificially using GM food (technology) to endanger the Chinese people. Based on this, this experiment was designed with three stimulus materials about genetically modified food (technology): neutral narratives, technology risk narratives, and conspiracy narratives. The conspiracy narratives focused on emphasizing intergroup conflicts of interest and vicious competition; the technology risk narratives highlighted the potential negative impacts of technology on human health, etc. In contrast, the neutral narratives served as a control group to present GM food or technology from an objective, non-specific narrative bias.

In order to verify the validity of the experimental materials, firstly, master’s degree students majoring in psychology and Chinese language and literature were invited to professionally revise three reading materials with similar structure and word count. Then, 12 undergraduates were arranged to participate in the trial reading and collect feedback. Finally, the initiation effect was tested through a behavioral tendency self-assessment question, “Would you consume genetically modified foods?” A five-point scale was used, with “1” representing definitely would and “5” representing definitely would not. *Post hoc* multiple comparisons showed that the neutral narrative group had significantly lower behavioral tendency scores than the conspiracy narrative group (*p* < 0.001) and the technological risk narrative group (*p* < 0.01), confirming that the material manipulation was effective. The complete experimental materials for Study 1 are available in the Appendix.

### 3.3 Experimental procedures

Study 1 utilized a one-way between-subjects design with three levels of narrative frames (neutral narratives, technological risk narratives, and conspiracy narratives) as the independent variables. Subjects were randomly assigned to one of the three narrative frames to enter the survey experiment. After reading the experimental materials, subjects were asked to recall and describe what they had read, followed by answering the following self-assessment question items: (1) “After reading the above materials, what is your opinion of the safety of genetically modified foods?” A four-point scale, with “1” representing not worried and “4” representing very worried, was used to measure the respondents’ level of risk perception. (2) “After reading the above materials, what measures would you like the government to take regarding the management of genetically modified foods?” A five-point scale was used, with “1” representing a full promotion of GM foods in China and “5” representing a complete ban of GM foods in China. Although this is a single-item measure, we explicitly defined “policy expectation” as the public’s attitude toward the government’s adoption of a particular policy (e.g., “comprehensively promote,” “comprehensively prohibit”), which can reflect the public’s expectation toward the government more accurately and effectively. The higher the score, the higher the public’s risk perception level, and the more they want the government to adopt policies that strictly control social risks. Finally, subjects filled in demographic variables such as gender and age.

### 3.4 Results and discussion

#### 3.4.1 Maneuvering checks

As a manipulation test, we measured subjects’ knowledge of the risks of genetically modified (GM) foods (technology) after reading the material and the behavioral tendency self-assessment entry: “How much do you know about the risks of the presence of genetically modified (GM) foods?” A four-point scale was used, with “1” representing no knowledge at all and “4” representing excellent knowledge; “Would you consume genetically modified foods?” A five-level scale was used, with “1” representing definitely would and “5” representing definitely would not. One-way ANOVA results showed that the public’s knowledge of risk [*F*(2, 366) = 10.020, *p* < 0.001] and behavioral tendency [*F*(2, 366) = 7.386, *p* < 0.01] across different narrative frames exhibited statistically significant differences, proving the validity of our manipulation.

#### 3.4.2 Risk perception

First, we performed an independent samples *t*-test for gender using SPSS 26.0 software. The results showed that the female group (*M* = 2.42, SD = 0.658) had significantly higher levels of risk perception than the male group [*M* = 2.17, SD = 0.726, *t*(367) = −3.228, *p* < 0.01]. In addition, we also found significant differences in risk perception levels by specialty [*F*(3, 365) = 2.892, *p* < 0.05], with the social sciences group (*M* = 2.43, SD = 0.706) having significantly higher risk perception levels than the science and engineering group (*M* = 2.21, SD = 0.712). A one-way analysis of variance (ANOVA) was conducted using SPSS 26.0 software, and the results showed significant differences in public risk perceptions across narrative frameworks [*F*(2, 366) = 7.539, *p* < 0.01]. The highest level of public risk perception was found when a conspiracy narrative was used (*M* = 2.44, SD = 0.69), while the lowest level of public risk perception was found when a neutral narrative was used (*M* = 2.09, SD = 0.72). Furthermore, *post hoc* multiple comparison analyses found that conspiracy narratives elicited significantly higher levels of risk perception than technical risk narratives (*p* < 0.05) and neutral narratives (*p* < 0.001). However, the difference between technical risk narratives and neutral narratives was not significant (*p* = 0.062). In conclusion, H1 and H2 hold, i.e., conspiracy narratives elicited the strongest risk perceptions relative to other narrative frames.

#### 3.4.3 Policy expectations

We conducted independent samples t-tests and one-way ANOVAs for gender, household registration, and specialty, respectively, and found that none of the effects were significant. There were significant differences in the public’s policy expectations of the government under different narrative frameworks [*F*(2, 366) = 66.379, *p* < 0.001]. Among them, under the conspiracy narrative framework, the public’s expectation of the government to adopt strict regulatory policies was the highest (*M* = 3.308, SD = 0.78), while under the neutral narrative framework, the public’s expectation of the government to adopt strict regulatory policies was the lowest (*M* = 2.23, SD = 0.68). *Post hoc* multiple comparisons analyses showed that conspiracy narratives elicited significantly higher expectations for strict regulatory policies than technology risk narratives (*p* < 0.001) and neutral narratives (*p* < 0.001); there was no significant difference between technology risk narratives and neutral narratives (*p* = 0.637). In summary, H3 concluded that conspiracy theory narratives contribute to the public’s expectations of the government’s strict regulatory policies.

#### 3.4.4 Summary

The results of Study 1 indicate that conspiracy narratives significantly increase the public’s level of risk perception. This suggests that when confronted with social risks, the public is more susceptible to media messages due to their limited cognitive abilities, and conspiracy narratives are more likely to cause panic and anxiety among the public due to the inclusion of strong emotional appeals and attributional inferences (40, 42), thus exacerbating risk perception. Similarly, within the framework of conspiracy narratives, the public is more likely to expect the government to adopt strict and restrictive regulatory policies to control social risks. However, since the government and the public do not have precisely the same information, the government cannot always make policy preferences that are entirely “in line with public opinion.” Expectation failure theory suggests that public trust in government is influenced by whether government policies meet their expectations (43). When government behavior does not meet public expectations, it leads to a crisis of public trust in the government and a deterioration of the relationship between the government and the people. Therefore, Study 2 will build on Study 1 to further explore how policy expectations affect public trust in government.

## 4 Experiment 2: the mediating role of policy satisfaction

Study 2 builds on the findings of Study 1 to further explore the effects of policy orientation and enemy involvement on public policy satisfaction and government trust in the context of conspiracy narratives.

### 4.1 Subjects

The sample size required for the experiment was estimated according to G*Power 3.1. The effect size was set to *f* = 0.32 by *F*-test, with a significance level of 0.05, and 111 were needed to achieve a statistical test power of 0.80. Study 2 was based on the “Conspiracy Narratives” group from Study 1, and 119 valid questionnaires were eventually extracted, including Strictly Restricted* Involved with Enemy Countries (30); Strictly Restricted* Not Involved with Enemy Countries (27); Positively Promoted* Involved with Enemy Countries (30); and Positively Promoted* Not Involved with Enemy Countries (32), which basically meets the sample size requirement. Seventy-seven males out of 119 participants, or 64.7%, were male. 69.7% of the participants majored in science and technology, and most were from rural areas. Table 2 shows the demographic details of the subjects.

**Table 2:** Demographic information of subjects (study 2).

(*N* = 119)	*N*	%
Gender		
Male	77	64.70%
Female	42	35.30%
Household registration		
Countryside	50	42.00%
Cities and towns	33	27.70%
Small or medium size city	22	18.50%
Large cities	14	11.80%
Major		
Science and Engineering	83	69.70%
Social Sciences (Economics, Law and Education)	27	22.70%
Humanities (Literature, History and Philosophy)	9	7.60%
Art and Physical education	0	0.00%

### 4.2 Experimental material

The experimental materials for Study 2 were taken from real-world policy news headlines about genetically modified (GM) food (technology). The policy news headlines were first categorized into two types: promotional and restrictive. Within these two types of headlines, a further distinction was made between cases involving enemy countries and cases not involving enemy countries. Finally, four categories of stimulus material were developed: severely restrictive* involving an enemy country, severely restrictive* not involving an enemy country, positively promoting* involving an enemy country, and positively promoting* not involving an enemy country. Each category of stimulus material contained five news headlines (Cronbach *α* = 0.851).

The relationship between government and the public can be generalized in several ways, one unique way being the use of themes, symbols, and metaphors in images (60). Therefore, this study uses image projection to measure the public’s trust in government. Considering that public reliance on the government often precedes trust in the government (60), this paper searched for images indexed by “reliance.” Thirteen images metaphorically referring to “reliance” were selected for this paper. The experimental materials were processed uniformly, and the images were all black and white with the same grayscale, size, and font. The size ratio of the images is 4:3. In order to avoid interfering with the surface validity of the relationship between the government and the public, the images are labeled with “gov” and “public,” respectively. To avoid interference with the face validity of the government-public relationship, the two sides in the pictures are labeled with “gov” and “public,” respectively. We invited 60 college students to fill in the words they thought represented the relationship between “gov” and “public” in the pictures. Based on the test feedback, pictures with vague or obscure relationship metaphors were further eliminated, leaving four pictures as self-assessment items of government trust (Cronbach’s *α* = 0.611). The complete experimental materials for Study 2 are available in the Appendix.

### 4.3 Experimental procedures

This study utilized a 2 (policy orientation: strict restriction vs. active promotion) × 2 (enemy country involvement: enemy country involved vs. no enemy country involved) between-subjects experimental design. Upon entering the experimental session, participants were randomly assigned to four groups of policy news headlines, each of which implied a specific government policy position. After reading the news headlines, participants were asked to complete a satisfaction self-assessment on a 6-point scale, with “1” representing very dissatisfied and “6” representing very satisfied, to measure their policy satisfaction. Participants were then asked to rate four pictures that were metaphors for the relationship between the government and the public: “Taking into account the information provided in the previous reading materials and the news headlines, rate the extent to which the metaphorical meaning of each of the pictures matches a certain kind of relationship between the government and the public in your mind” on a 6-point scale in which “1” stands for very little conformity and “6” stands for very much conformity, as a measure of subjects’ trust in the government. The above self-assessment entries were used as dependent variables, with higher scores indicating higher public policy satisfaction and government trust.

### 4.4 Results and discussion

#### 4.4.1 Maneuvering checks

ANOVA results showed that subjects were significantly more satisfied with the policy in the strict restriction group (*M* = 4.572, SD = 0.113) than in the positive promotion group (*M* = 3.177, SD = 0.108), *F*(1, 117) = 79.782, *p* < 0.001, =0.410, indicating that the experimental manipulation was valid.

#### 4.4.2 Policy satisfaction

**Figure 2:**
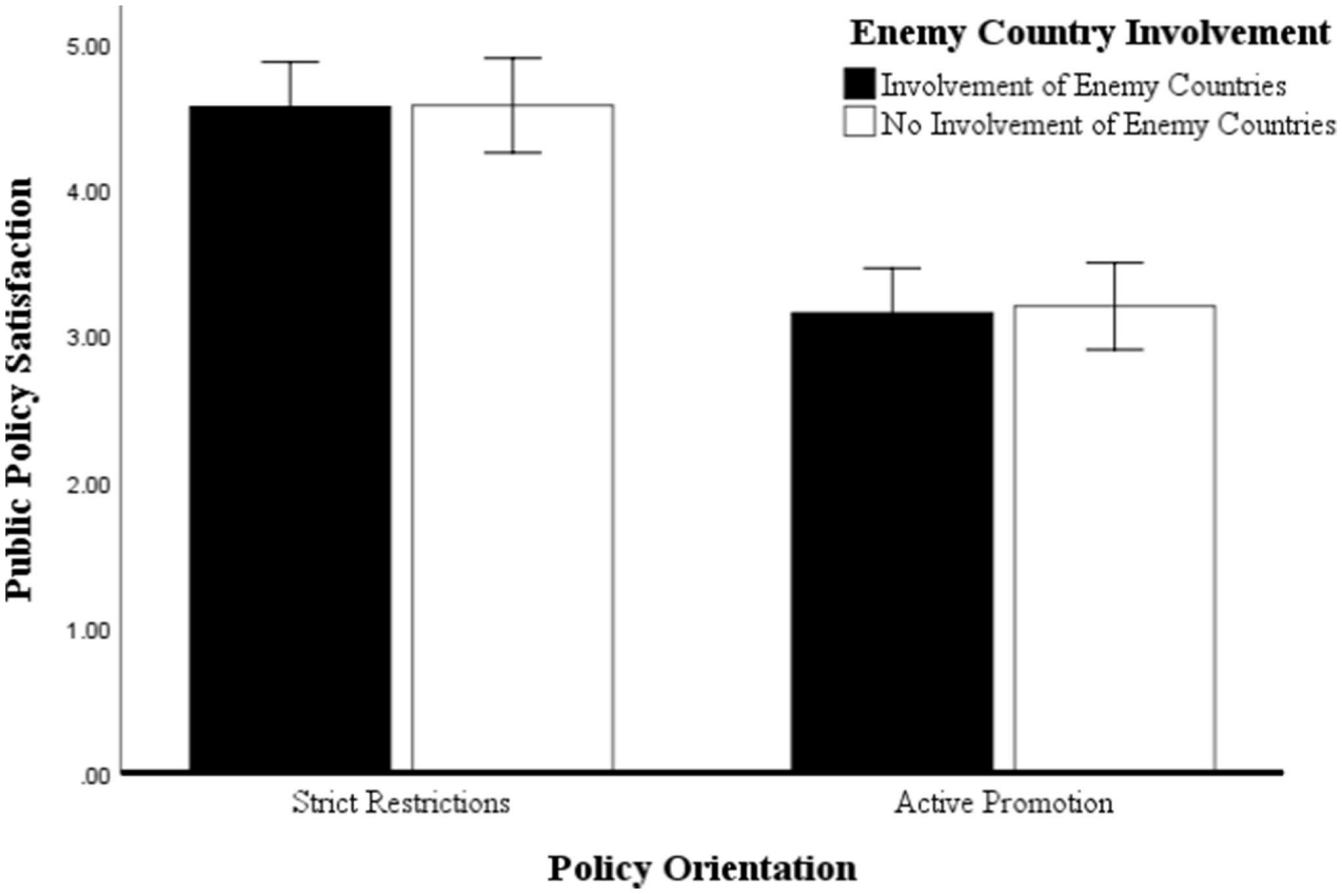
ANOVA for policy satisfaction.

Study 2 conducted a two-way ANOVA with main and interaction effects using SPSS 26.0. Regarding policy satisfaction, the results are shown in Figure 2, where the main effect of policy orientation was highly significant. Policy satisfaction was significantly higher in the severely restrictive group (*M* = 4.572, SD = 0.779) than in the actively promoting group (*M* = 3.177, SD = 0.899), *F*(1, 117) = 79.782, *p* < 0.001, =0.410; the main effect of enemy participation was not significant, with the participation in the enemy group (*M* = 3.860, SD = 1.109) and the non-participation in the enemy group (*M* = 3.831, SD = 1.086) differed insignificantly, *F*(1, 117) = 0.034, *p* = 0.854. The interaction between policy orientation and enemy involvement was similarly insignificant, *F*(1, 117) = 0.013, *p* = 0.910. Notably, although the interaction effect was insignificant, the data results suggest that in the conspiracy narrative approach, when the government adopts actively promoted policies and the news headlines involve enemy countries, public policy satisfaction is lowest; when the government adopts strictly limited control policies, and the news headlines involve enemy countries, public policy satisfaction tends to exceed the satisfaction when enemy countries are not involved progressively.

#### 4.4.3 Government trust

The same independent samples *t*-test was used to explore gender differences in government trust, which showed that the male group (*M* = 3.989, SD = 0.805) had significantly higher government trust than the female group [*M* = 3.464, SD = 0.796, *t*(117) = 3.413, *p* < 0.01]. The results of one-way ANOVA and *post hoc* multiple comparisons, on the other hand, showed significant differences in government trust across specialties [*F*(2, 116) = 9.581, *p* < 0.001], with the science and engineering group (*M* = 4.01, SD = 0.781) having a significantly higher level of trust in the government than the social sciences group (*M* = 3.355, SD = 0.764) and the humanities group (*M* = 3.25 SD = 0.848). Whereas, the place of domicile did not have a significant effect on the subjects’ trust in government.

**Figure 3:**
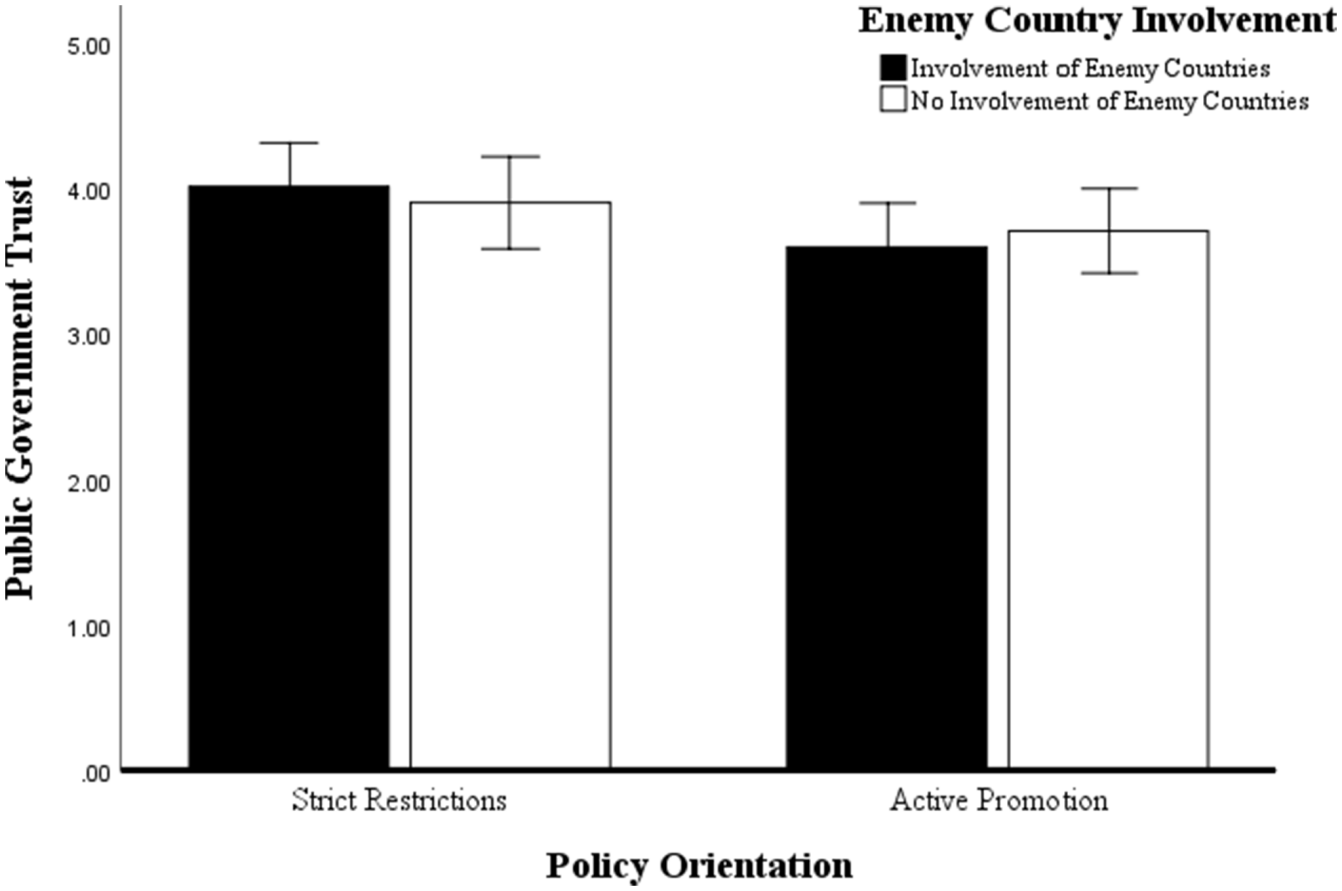
ANOVA of government trust.

Regarding government trust, the results are shown in Figure 3, with a significant main effect of policy orientation. Trust in government was significantly higher in the severely restrictive group (*M* = 3.963, SD = 0.856) than in the actively promoted group (*M* = 3.644, SD = 0.797), *F*(1, 117) = 3.981, *p* < 0.05, $\eta_{p}^{2}$ = 0.033. The main effect of hostile country involvement was not significant. The difference between the group involved in a hostile country (*M* = 3.808, SD = 0.834) and the not involved in a hostile country group (*M* = 3.780, SD = 0.848) did not differ significantly, *F*(1, 117) = 0, *p* = 0.997. In addition, the interaction between policy orientation and enemy countries involvement was similarly insignificant, *F*(1, 117) = 0.531, *p* = 0.467. In summary, Hypothesis 4 was valid, and Hypothesis 5 was not valid. Under the conspiracy narrative, policy orientation significantly affects the public trust in the government, which is higher when the government is perceived to be practicing severely restrictive control policies.

**Figure 4:**
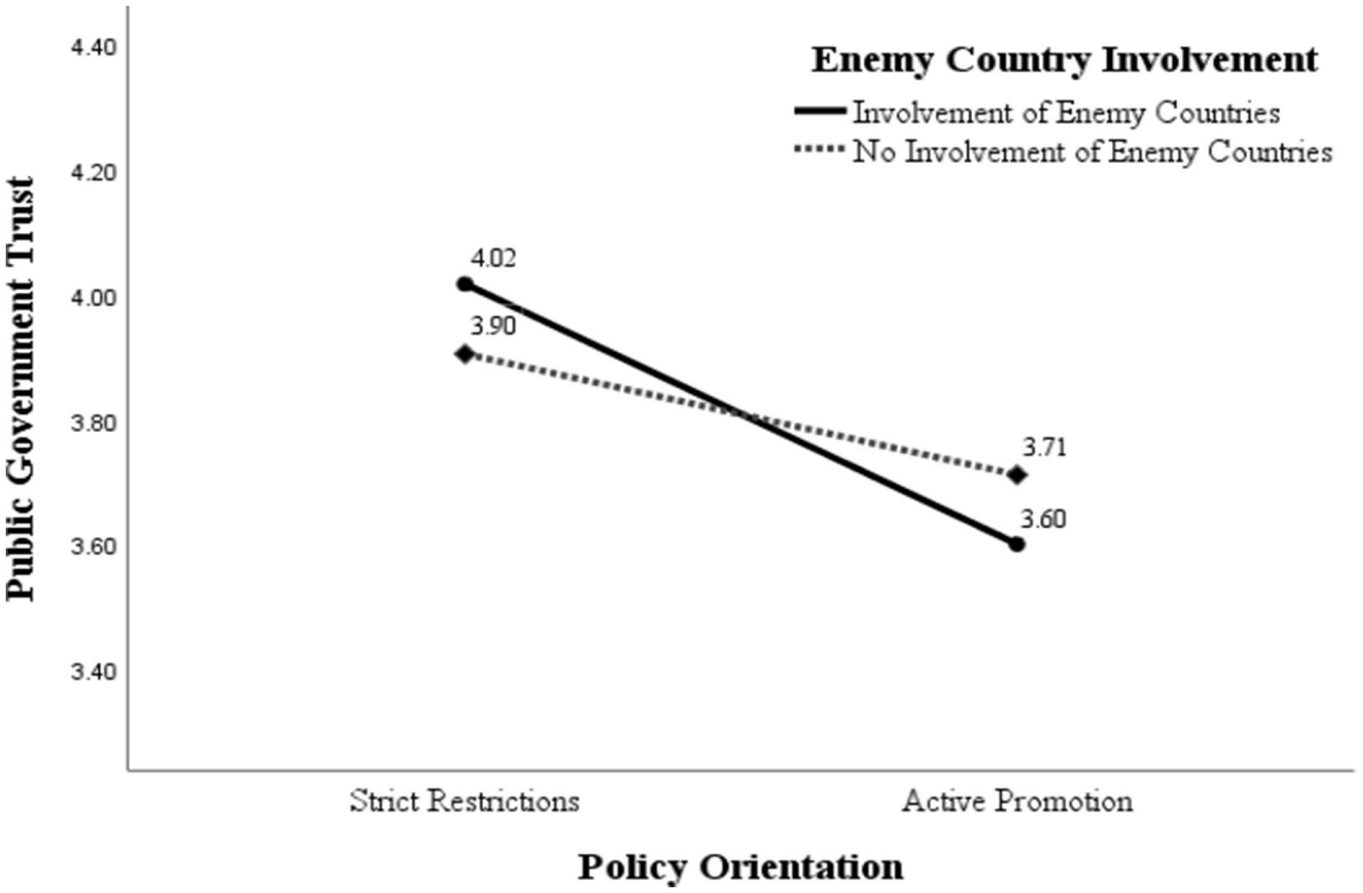
Simple effect of the interaction of policy orientation and enemy countries involvement on trust in government.

Although the interaction between the two variables was not significant, there was a tendency for the interaction effect to be significant in the study. Under the conspiracy narratives, strict restriction* involves enemy countries group (*M* = 4.016, SD = 0.890); strict restriction* does not involve enemy countries group (*M* = 3.904, SD = 0.829); active promotion* involves enemy countries group (*M* = 3.600, SD = 0.729); and active promotion* does not involve enemy countries group (*M* = 3.711, SD = 0.866). It can be seen that the public trust in the government is highest when the government adopts a strictly limited control policy and enemy countries are involved in the news. In contrast, the public trust in the government is lowest when the government adopts an active promotion policy, and enemy countries are involved, as shown in Figure 4.

#### 4.4.4 The mediating role of policy satisfaction

**Figure 5:**
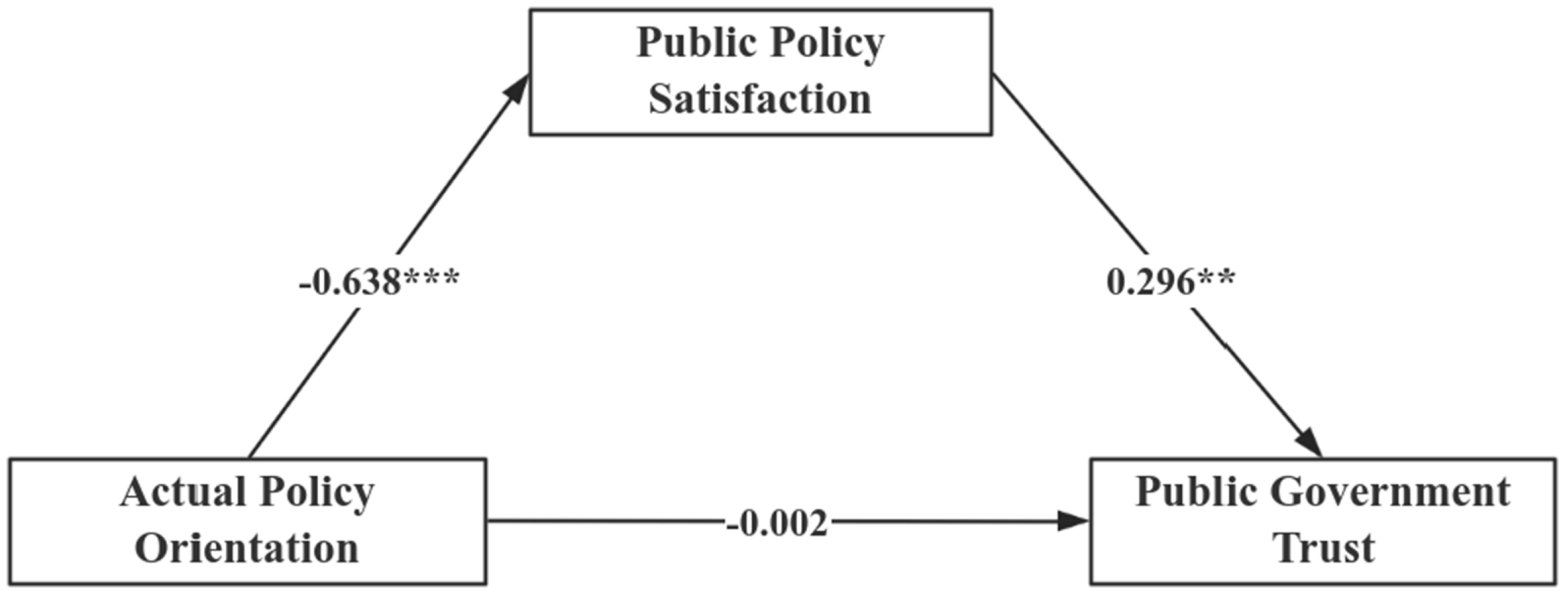
Inter mediation effect path diagram.

To further understand the role of policy satisfaction in the relationship between policy orientation and government trust, we utilize Model 4 of the PROCESS macro to test the mediating effect of policy satisfaction. As shown in Tables 3, 4, the results indicate that government policy orientation indirectly affects government trust through policy satisfaction (*β* = 0.296, *p* < 0.01, LLCI = −0.630, ULCI = −0.012). Specifically, under the conspiracy narratives, the public level of risk perception is elevated, and the expectation of the government to adopt strict control policies is more substantial, so when the public perceives the strictly limited control policies adopted by the government, their policy satisfaction increases and government trust increases. In summary, hypothesis H6 holds that policy satisfaction fully mediates the relationship between policy orientation and government trust (as shown in Figure 5), and public policy expectations play a moderating role between government policy orientation and public policy satisfaction.

**Table 3:** Mediating effects path analysis.

Trails	Effect	SE	LLCI	ULCI
Direct effect of X on Y	−0.003	0.218	−0.460	0.401
Indirect effect of X on Y	−0.316	0.159	−0.630	−0.012
Total effect of X on Y	−0.320	0.152	−0.621	−0.019

**Table 4:** Regression analysis of mediating effects.

Variables	Public government trust		Public policy satisfaction		Public government trust	
	*β*	*t*	*β*	*t*	*β*	*t*
Actual policy orientation	−0.191*	−2.103	−0.638***	−8.954	−0.002	−0.017
Public policy satisfaction					0.296**	2.574
*R* ^2^	0.036		0.407		0.089	
*F*-value	4.424*		80.177***		5.632**	

*n* = 119. *p < 0.05, **p < 0.01, ***p < 0.001.

The results of Study 2 indicate that government policy orientation significantly affects public policy satisfaction and government trust. Specifically, under the conspiracy narratives, if the public perceives that the government implements a strictly restrictive control policy on genetically modified (GM) foods, the public trust in the government increases; conversely, if the public perceives that the government implements a policy that actively promotes GM foods, the public trust in the government decreases. In this process, public policy satisfaction acts as a complete mediator, and public policy expectations mediate between government policy orientation and public policy satisfaction.

## 5 Conclusion and recommendations

### 5.1 Main findings

This study reveals three core findings. First is the differential effect of narrative framing. By enhancing perceptions of technological risk, conspiracy narratives significantly increase public support for restrictive policy claims over and above mere technological risk narratives. At this point, conspiracy theories, like populist rhetoric, can undermine trust and trigger extreme public reactions (55).

Second, the dual-path regulation mechanism of policy expectations. It reveals that the dynamics of public trust in government is affected by the degree of fit between their policy expectations and actual policy preferences. The findings show that the match between policy and public expectations increases government trust and vice versa. Meanwhile, policy satisfaction plays a fully mediating role in forming institutional trust, a finding that supports the applicability of expectation failure theory in authoritarian settings (46).

Finally, the mechanisms by which risk communication shapes trust in government are constrained by multiple non-narrative elements. The boundaries of the effectiveness of risk communication need to be examined in the context of a larger governance framework. Interestingly, there is no statistically significant interaction between restrictive policies and the involvement of enemy forces in influencing public trust. This may stem from other factors, such as socioeconomic factors, fairness of governance, or pre-existing public perceptions of the government, which may be more influential in shaping trust than narratives associated with enemy powers (50, 51).

### 5.2 Recommendations

With the proliferation of new media, the accessibility of conspiracy narratives to the general public, particularly those lacking critical media literacy, poses significant challenges for risk communication. Intergroup conspiracy narratives have exacerbated public concerns over emerging technologies, such as GM foods, often leading to disproportionate support for restrictive policies that hinder technological development and societal progress. For instance, conspiracy narratives surrounding vaccines and 5G technology—such as claims that vaccines are tools for population control or that 5G signals spread viruses—have triggered public panic and irrational behaviors (8, 9). These narratives distort scientific objectivity and erode public trust in government, potentially undermining effective policy implementation and technological advancement (20). To address these challenges, governments should adopt proactive strategies to counter conspiracy narratives and enhance trust through effective risk communication.

#### 5.2.1 Precise and efficient media literacy improvement strategies

Research has shown that the interaction between risk perception and policy expectations suggests creating a media literacy framework integrating education, communication, and participation (38). Specific measures are as follows: first, the education system should incorporate the cultivation of information recognition skills, focusing on identifying the characteristics of conspiracy theories (e.g., extremist rhetoric, insufficient evidence) and analyzing the relevance of the interests of the information sources; then, the mainstream media needs to play its guiding role in shaping rational public opinion, motivating the public to keep questioning the spirit of false information, and establishing sound and transparent communication channels; and, finally, constructing a transparent communication system covering technical hearings, citizens’ juries and rumor reporting platforms, and regularly publicize the professional evaluation process of important scientific and technological decisions. Throughout the implementation process, special attention must be paid to cultural adaptation.

#### 5.2.2 Precision response strategies for proactive intervention

When conspiracy narratives simultaneously possess significant public harm, broad dissemination, and government credibility relevance, the government should activate a multidimensional proactive intervention mechanism. This study confirms that conspiracy theories involving primary public interests, such as GM food safety, will lead to a decline in public trust if they spread too much and directly question government credibility. In this regard, it is recommended to implement the “scientific-emotional-institutional” three-dimensional response: in the scientific dimension, the original experimental data will be published within 72 h, and the credibility can be enhanced through visual evidence; in the emotional dimension, the organization of the directly affected groups to speak out, which has a higher effect than the officials’ clarification; in the institutional dimension, the opening up of the third-party testing application channel to repair the damage to the trust of the integrated interventions need to grasp the golden 72-h window, after which the effect will diminish. If the window is exceeded, the effect will diminish.

#### 5.2.3 Risk control strategies for silent management

Strategic silence is more effective in conjunction with implicit de-escalation for low-risk conspiracy theories that spread within specific subcultural circles and do not have a clear policy agenda. When the spread is confined to a specific online community, without an apparent policy demand, and the rebuttal may trigger a reverse effect, direct intervention may expand the scope of the spread (56). In this case, it is recommended to use algorithmic silent processing, such as reducing the weight of the topic hot search, combined with attention diversion, through the push of more attractive alternative content to realize the natural fading. However, it is necessary to establish a continuous monitoring mechanism and immediately launch an emergency response when the propagation breaks through the threshold. This hierarchical management strategy not only avoids the waste of resources but also prevents the boomerang effect, which is in line with the law of marginal utility of risk communication.

## 6 Summary

### 6.1 Findings and significance

This study emphasizes that conspiracy narratives significantly erode government trust by reinforcing the discrepancy between public risk perceptions and policy expectations. Further, when actual policy preferences match the public’s policy expectations, trust in government is enhanced, and vice versa, and a crisis of trust is exacerbated.

This study realizes a double breakthrough at the theoretical and practical levels. At the methodological level, it innovatively adopts image projection technology to deconstruct the representation of government-citizen interaction through visual symbols, breaking through the social approval bias of traditional measurement. At the theoretical level, a transmission model of “conspiracy narrative – risk perception – policy expectation – government trust” is constructed, which reveals the mediating role of policy satisfaction on institutional trust and makes up for the limitations of traditional research on the influence mechanism of policy trust. At the practical level, this study suggests that the government should measure conspiracy narratives differently according to the public’s risk perception and policy expectations. The research results provide an interdisciplinary solution for the paradigm shift of government-citizen interaction in the era of technological governance, realizing the construction of a dynamic governance path from risk perception to government trust.

### 6.2 Research limitations and future directions

Two limitations are worth noting. On the one hand, this study reveals that the transmission mechanism of “narrative frame-policy expectation-government trust” is cross-culturally applicable, but its effects are affected by regional characteristics. Therefore, future research should examine the semantic differences in cultural transplantation (27). In collectivist cultures, such as East and Southeast Asia, increasing power distance may enhance the link between policy preferences and government trust; in liberal societies, such as Europe and the United States, attention should be paid to adjusting narrative dimensions and emphasizing the symbolic significance of procedural justice and public participation. In geopolitically conflict-prone regions, such as Central and Eastern Europe, attention should be paid to the sensitivity of the hostile state element in conspiracy narratives.

On the other hand, the study results are specific to China, with college students as the primary sample, which limits the generalizability of the results. Future research should include more diverse cultural and demographic backgrounds. Meanwhile, this study focused on trust as a precursor to behavior, but the relationship between trust and actual behavior deserves further research. Future research could build on the chain model of this study and conduct cross-technology comparative experiments. For example, comparing the “government-drug company” trust game in the field of vaccines with the “expert-public” risk discounting model in nuclear energy. Such an extension can not only verify the theoretical boundaries but also construct a typological framework of technology governance, which is the focus of our subsequent research.

## Data Availability

The original contributions presented in the study are included in the article/Supplementary material, further inquiries can be directed to the corresponding author.
