# Supplementary material for: How risk communication affects public trust in government: the moderating role of policy expectations
Source: Front Public Health. 2025 May 14;13:1557786. doi: 10.3389/fpubh.2025.1557786 (PMC12116495; doi:10.3389/fpubh.2025.1557786)
Supplement: Supplementary file 2 [file Presentation_1.pdf]

Supplementary Material

Attitude Survey on Genetically Modified Food

Hello! In order to know the public's attitude towards genetically modified (GM) food, we have conducted this questionnaire survey. Please fill in the following questions carefully according to your true thoughts and check the corresponding option numbers. The information you provide will only be used for scientific research and will be kept strictly confidential, so please feel free to answer the questions. Thank you very much for your cooperation and support!

I. Basic information:

A1. Sex: 1. male 2. female

A2. Household registration: 1. rural 2. urban 3. small and medium-sized cities 4. large cities

A3. Current major: 1. science and technology 2. social sciences 3. humanities 4. arts and sports

II.Please read the following passages about genetically modified food carefully before answering the following questions.

Passage A

Since the 20th century, genetically modified (GM) technology has enabled humans to cross natural species barriers, reshaping organisms according to their desires. With commercialization, GM technology has moved out of the laboratory into everyday life, resulting in a proliferation of GM foods on the market. Advocates claim that GM foods can address food shortages in developing countries. However, critics warn that large-scale commercialization of GM foods could become a covert form of warfare, particularly against developing nations.The global development of GM technology is marked by significant disparities, with research and production dominated by developed countries. This monopolization poses threats to food sovereignty in developing nations, potentially allowing powerful countries to control food supplies. Critics argue that promoting GM food commercialization is part of a hidden agenda, with the populations of developing countries serving as "guinea pigs" to test the safety of GM technology. Some scholars and officials have suggested that GM foods could function as control tools, likening them to a weapon more potent than nuclear arms. To counter this, national leaders emphasize the importance of strengthening biosecurity and safeguarding food sovereignty.

Passage B

Genetically modified (GM) food is a product of advanced biotechnology but carries significant potential risks. Critics suggest that humanity is venturing into agricultural biotechnology with high expectations but little regulation and insufficient understanding of long-term consequences.The risks associated with GM foods include the potential for foreign genes to disrupt food nutrients in unpredictable ways and the lack of long-term safety testing. Even if GM foods appear safe now, their

long-term impacts remain uncertain. Additionally, GM foods may trigger allergic reactions. For example, transferring genes from corn to soybeans, wheat, or shellfish may introduce allergens into these foods. Another example is soybean oil made from GM soybeans containing a saturated fat gene, which may increase the risk of cardiovascular diseases, fatty liver, or high cholesterol due to the formation of saturated fats in the body.

Passage C

Genetically modified (GM) technology allows scientists to modify organisms by transferring specific genes from one species to another. This process, known as transgenic technology, enables the creation of genetically modified organisms (GMOs) with new traits, such as higher yields, improved quality, and increased resistance to pests, diseases, or environmental stressors.Foods derived from GMOs, such as GM soybeans and their byproducts (e.g., soybean oil, tofu, soy sauce, and tempeh), are considered GM foods. The theoretical foundation of GM technology lies in molecular biology, which enables the synthesis and integration of specific DNA fragments into target organisms. These organisms are then selectively bred over several generations to produce stable new species with desired traits. This innovation has the potential to transform agriculture and food production significantly.

B1. After reading the above materials, to what extent do you understand the risks of genetically modified foods?

1 2 3 4

Not at all Somewhat aware Comparatively aware Very aware

B2. After reading the above materials, how do you feel about the safety of genetically modified foods?

1 2 3 4

Not at all worried Somewhat worried Quite worried Very worried

B3. After reading the above materials, would you consume genetically modified food? (degree of rejection)

1. definitely would 2. tend to would 3. not sure 4. tend not to would 5. definitely would not

B4. After reading the above materials, what do you expect the Government to do about the management of genetically modified food?

1. Make every effort to promote the development of genetically modified foods in China.
2. Promote the development of genetically modified foods in China in a prudent manner.
3. No expectation of government action and a wait-and-see attitude.

4. Strictly limiting the development of genetically modified foods in China.
5. A total ban on genetically modified foods in China.

III.The following are five recent news headlines related to genetically modified (GM) food (crops), which may reflect the government's policy attitude towards GM food. Combined with the previous reading materials, please evaluate the feelings brought to you by each news headline, with "1" indicating that you are "very dissatisfied" with the information hidden in this news headline, and "6" indicating that you are "very satisfied" with the information hidden in this news headline. 1" means you are "very dissatisfied" with the message hidden in the headline, and "6" means you are "very satisfied" with the message hidden in the headline. **Please tick or circle the appropriate number.**

News headlines (a1b1)	It's very unsatisfactory	unsatisfactory	unsatisfactor y	mostly satisfactory	dissatisfied	very happy
**Complicated approval process for** GM products from **hostile countries** to enter China, subject to hundreds of safety assessments	1	2	3	4	5	6
China Agriculture Office: China's GM market **cannot be occupied by hostile countries**	1	2	3	4	5	6
China **rejects** genetically modified corn from hostile countries, returns more than 10 billion tons of shipments	1	2	3	4	5	6
The Chinese government **has suspended imports of** a rival country's genetically modified soybeans based on domestic public opinion.	1	2	3	4	5	6
China's agriculture minister: **do not allow** GM technology to be monopolized by hostile countries	1	2	3	4	5	6

News headlines (a1b2)	It's very unsatisfactory	unsatisfactory	unsatisfactory	mostly satisfactory	dissatisfied	very happy
Ministry of Agriculture: opening of online reporting, supervision and investigation, no spread of illegal cultivation of genetic modification	1	2	3	4	5	6

The Ministry of Agriculture notified the "genetic modification testing center falsification" and the deadline for rectification	1	2	3	4	5	6
Four provinces were interviewed by the Ministry of Agriculture (MOA) for failing to supervise the illegal cultivation of genetically modified (GM) plants.	1	2	3	4	5	6
Circular of the General Office of the Ministry of Agriculture on the Handling of 11 Units Violating Agricultural Genetically Modified Organism Safety Management Regulations	1	2	3	4	5	6
State Council Legislative Affairs Office: genetically modified food should be significantly labeled, food safety implementation of the three recalls	1	2	3	4	5	6

**News headlines (a2b1)**	**It's very unsatisfactory**	**unsatisfactory**	**unsatisfactory**	**mostly satisfactory**	**dissatisfied**	**very happy**
Ministry of Agriculture: vigorously promoting cooperation with hostile countries to advance the commercialization of transgenic rice	1	2	3	4	5	6
China agrees to import genetically modified corn from hostile countries	1	2	3	4	5	6
Sino-Foreign Agricultural Investment and Cooperation Forum: Hot Topics on Cooperation in Promoting Genetically Modified Crops	1	2	3	4	5	6
Ministry of Agriculture: Sino-Foreign Agricultural Cooperation Shows Very Favorable Trend	1	2	3	4	5	6
According to relevant media reports, China currently imports nearly 100 million tons of genetically modified soybeans from hostile countries	1	2	3	4	5	6

**News headlines (a2b2)**	**It's very unsatisfactory**	**unsatisfactory**	**unsatisfactory**	**mostly satisfactory**	**dissatisfied**	**very happy**
China becomes world's first country to approve staple grains for GMO cultivation	1	2	3	4	5	6
Central Agricultural Office: GM commercial planting is expanding internationally, China can't lag behind	1	2	3	4	5	6

Ministry of Agriculture: Unspecified variety approval should not be a "roadblock" to China's GM industrialization	1	2	3	4	5	6
Ministry of Agriculture: the promotion of genetically modified food will not be shaken by the "reported testing center incident".	1	2	3	4	5	6
Ministry of Agriculture: China must actively promote genetically modified foods	1	2	3	4	5	6

IV. Below are 11 statements about the regulation of genetically modified (GM) foods. Based on the information you have obtained from the previous reading materials and the news headlines, please rate how much you agree with the following statements, with "1" representing "strongly disagree", "6" representing "Strongly Agree", and 2, 3, 4 and 5 are between the two extremes. **Please tick or circle the appropriate number.**

	**Strongly disagree**	**disagree**	**Not really**	**basic agreement**	**agree with**	**couldn't agree more**
Information released by the government relating to genetically modified food is true and reliable	1	2	3	4	5	6
Government departments will fully consider the interests of the public when formulating policies related to genetically modified foods	1	2	3	4	5	6
The Government will select scientists impartially and objectively to provide professional advice on GM food decisions	1	2	3	4	5	6
Government departments have no self-interest in formulating policies on genetically modified foods	1	2	3	4	5	6

V. The pictures have a metaphorical meaning. Combined with the information provided in the previous reading materials and news headlines, evaluate the degree to which the metaphorical meaning of each picture corresponds to some kind of relationship between the government and the public in your mind, with “1” standing for “very inconsistent”, “6” for “very inconsistent”, and 2, 3, 4, 5 falling between the two extremes. “1” means ‘not at all’, ‘6’ means ‘very much’, and 2, 3, 4, and 5 are in between the two extremes. **Please tick or circle the appropriate number.**

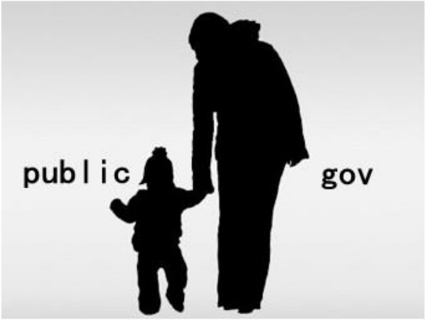

1 2 3 4 5 6

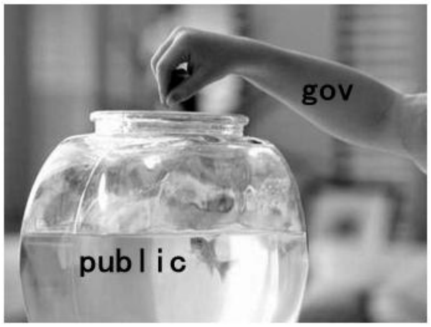

1 2 3 4 5 6

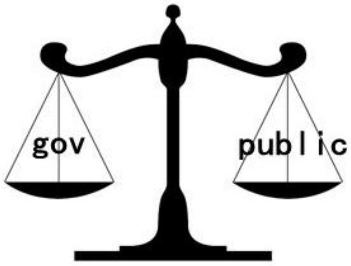

1 2 3 4 5 6

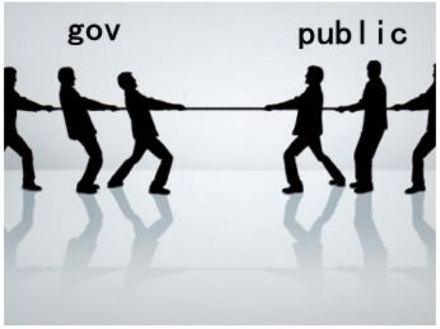

1 2 3 4 5 6

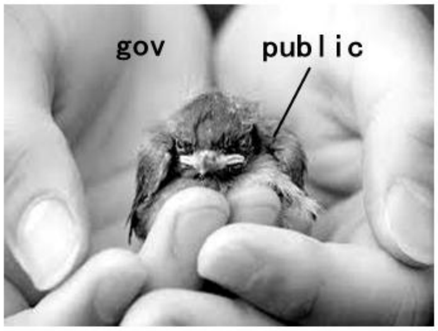

1 2 3 4 5 6

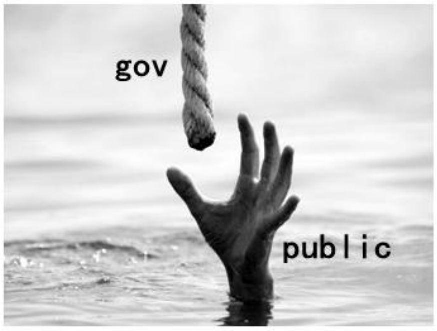

1 2 3 4 5 6
